# Pristane-Induced Arthritis Loci Interact with the *Slc11a1* Gene to Determine Susceptibility in Mice Selected for High Inflammation

**DOI:** 10.1371/journal.pone.0088302

**Published:** 2014-02-05

**Authors:** Marcelo De Franco, Luciana C. Peters, Mara A. Correa, Antonella Galvan, Tatiane Canhamero, Andrea Borrego, José R. Jensen, Jussara Gonçalves, Wafa H. K. Cabrera, Nancy Starobinas, Orlando G. Ribeiro, Tommaso Dragani, Olga M. Ibañez

**Affiliations:** 1 Laboratório de Imunogenética, Instituto Butantan, São Paulo, Brazil; 2 Department of Experimental Oncology, Istituto Nazionale dei Tumori, Milan, Italy; National Institute of Allergy and Infectious Diseases, United States of America

## Abstract

AIRmax (maximal inflammation) and AIRmin (minimal inflammation) mice show distinct susceptibilities to pristane-induced arthritis (PIA). The *Slc11a1* gene, which regulates macrophage and neutrophil activity, is involved in this infirmity. AIRmax*^SS^* mice homozygous for the non-functional *Slc11a1 S* (gly169asp) allele obtained by genotype-assisted crosses from AIRmax and AIRmin mice are more susceptible than mice homozygous for the *Slc11a1* resistant (*R*) allele. The present work sought to identify the quantitative trait loci (QTL) regulating PIA and to examine the interactions of these QTL with *Slc11a1* alleles in modulating PIA. Mice were given two ip injections of 0.5 mL pristane at 60 day intervals, and the incidence and severity of PIA was scored up to 160 days. Genome-wide linkage studies were performed to search for arthritis QTL in an F2 (AIRmax × AIRmin, n = 290) population. Significant arthritis QTL (LODscore>4) were detected on chromosomes 5 and 8, and suggestive QTL on chromosomes 7, 17 and 19. Global gene expression analyses performed on Affymetrix mouse 1.0 ST bioarrays (27k genes) using RNA from arthritic or control mice paws showed 419 differentially expressed genes between AIRmax and AIRmin mice and demonstrated significantly (P<0.001) over-represented genes related to inflammatory responses and chemotaxis. Up-regulation of the chemokine genes *Cxcl1*, *Cxcl9*, *Cxcl5*, *Cxcl13* on chromosome 5 was higher in AIRmax*^SS^* than in the other lines. *Macrophage scavenger receptor 1* and *hemeoxigenase (decycling) 1* genes on chromosome 8 were also expressed at higher levels in AIRmax*^SS^* mice. Our results show that the gene expression profiles of the two arthritis QTL (on chromosomes 5 and 8) correlate with *Slc11a1* alleles, resulting in enhanced AIRmax*^SS^* mice susceptibility to PIA.

## Introduction

Mouse lines phenotype-selected for the maximum (AIRmax) or minimum (AIRmin) acute inflammatory reactivity (AIR) were used to study the impact of the genetic control of nonspecific immunity on susceptibility to autoimmune [1], neoplastic [2], and infectious diseases [3]. AIRmax and AIRmin mice were developed through bidirectional selection, starting from a highly polymorphic population (F0) derived from the intercrossing of eight inbred mouse strains (A, DBA2, P, SWR, CBA, SJL, BALB/c and C57BL/6). The selection phenotypes chosen were localized leukocyte influx and exudated plasma proteins 24 hr after the subcutaneous injection of polyacrylamide beads (Biogel), a non-antigenic, insoluble, and chemically inert substance [4]. The progressive divergence of the AIRmax and AIRmin lines during successive generations of selective breeding reached 20- and 2.5-fold differences in leukocyte infiltration and exudated protein concentrations respectively. These differences resulted from the accumulation of alleles in quantitative trai loci (QTL) endowed with opposite and additive effects on the inflammatory response. We can consider AIRmax and AIRmin as outbred stock mice derived from eight inbred lines and submitted to extensive bidirectional selective process for strong and weak acute inflammation phenotypes. Inbreeding was avoided for the selective breeding, as such AIRmax and AIRmin mice maintain a heterogeneous genetic background but are homozygosus in acute inflammation modifier loci. Analysis of the selective processes indicated that the AIR phenotype is regulated by at least 11 QTL [2].

The acute inflammation response to Biogel, as well as susceptibility to pristane induced-arthritis [5], to *S.* enterica serotype Typhimurium infection, and to the LPS of the bacteria were all modified in these mice; and linkage analysis with microsatellite markers mapped QTL in chromosomes 1, 6, and 11 which are relevant to these phenotypes [6].

Alterations in bone marrow granulopoiesis in response to hematopoietic factors and the production of chemotactic factors by infiltrated or local resident cells both contribute to phenotypic differences between the two lines. Convergent phenotypes in AIRmax mice were observed that were characterized by high neutrophil production in bone marrow, a high number of neutrophils in the blood, high concentrations of chemotatic agents, and increased resistance of infiltrating neutrophils to spontaneous apoptosis [7].

Tissue repair was also investigated in these two lines, revealing that AIRmax mice present a high regeneration capacity in comparison to AIRmin mice. Inflammatory QTL on chromosomes 1 (*Slc11a1* gene region) and 14 were found to regulate tissue regrowth in this model [8]. Additionally, the same chromosome 1 QTL seems to regulate leukocyte and protein influx during acute inflammation, as well as arthritis incidence and severity [5].

Rheumatoid arthritis (RA) affects approximately 0.5 to 1% of the world population [9] and is modulated by immune and inflammatory processes, as well as by class II MHC (in particular the locus HLA-*DRB1*) [10], non-MHC genes [11] and environmental factors. Among RA mouse models, pristane-induced arthritis (PIA) [12] resembles the human condition by its chronic inflammatory nature, similar histopathology and dependency of specific immunity, in particular CD4+ T cells [13]. Another feature of PIA in the mouse is the requirement of microbiota stimulation [14].

Pevious studies have shown that AIRmax mice are extremely susceptible to PIA, whereas AIRmin mice are resistant [1]. The incidence and severity of PIA in AIRmax mice is similar to that of inbred DBA/1 mice [15], but is more intense than those seen in BALB/c [12] or CBA Igh^b^ [16] mice. Fifteen to 25% of the susceptible inbred individuals in lines such as BALB/c [12] and CBA Igh^b^ [17] developed arthritis 200 days after pristane injection. Specific pathogen-free CBA Igh^b^ mice do not develop this disease, but will develop arthritis upon transfer to a conventional environment in the same way as CBA Igh^b^ mice raised conventionally since birth – indicating the involvement of environmental factors in PIA [14]. Susceptibility to PIA is CD4+ T cell (Th) dependent [13] and has been associated with increased agalactosylIgG levels mediated by IL6 production [18], while protection against PIA is mediated by Th2-associated cytokines produced after hsp65 pre-immunization [19].

In contrast to the immune response profile observed in inbred mice, Th2-type response high IgG1 anti-hsp65 levels and high numbers of IL4, IL6, and TNFa secreting splenic cells were observed in susceptible AIRmax mice, following pristane treatment. On the other side, Th1-dependent IgG2a was the predominant isotype in resistant AIRmin mice and increased IFNg producing cells in spleen was evident in these mice only [1].

*SOLUTE CARRIER FACTOR 11 A 1 (SLC11A1)* gene polymorphism has been implicated in human RA susceptibility [20]. In the mouse, *Slc11a1* codes for a transport protein expressed at the membrane of macrophage phagosomes [21] and it is associated with the transport of essential ions. This gene has been described in mice as a major modulator of susceptibility to infectious diseases (first named *Natural resistance associate macrophage protein 1* (*Nramp1*) gene) [22], and is expressed in macrophages and neutrophils [23]. *Slc11a1* is pleiotropic, interfering with macrophage activation, oxidative and nitrosamine bursts [24], TNFα, IFNγ, and IL-1 production [25], and the expression of MHC class II molecules [26]. The mutation corresponding to the *Slc11a1 S* (susceptibility) allele determines a gly169asp substitution resulting in a non-functional protein [27] that promotes ion accumulations inside the phagosome that favor pathogen replication [28].

In the selection process for AIRmax and AIRmin mouse lines, the *Slc11a1 S* allele frequency was 25% in the founder population (F0), but shifted to 60% in AIRmin and to 9% in AIRmax after 30 generations of selective breeding for inflammatory response, suggesting that these frequency changes were, in fact, the result of genetic selection [3]. In order to determine if the accumulation of *Slc11a1* alleles in the AIR lines was due to selection of inflammation QTL and not a chance event, *Slc11a1* homozygous mice for the susceptibility *(S)* or resistant *(R)* alleles with either AIRmax or AIRmin genetic backgrounds were produced through genotype-assisted mating. The resulting sublines were designated AIRmax*^RR^*, AIRmax*^SS^*, AIRmin*^RR^*, and AIRmin*^SS^*. The interaction of the *Slc11a1 S* allele with high inflammatory background QTL in AIRmax mice was evident through the down-modulation of Biogel-induced early acute inflammation and resistance to *Salmonella* Typhimurium infection [6], as well as by the aggravation of pristane-induced arthritis [5].

We recently identified several acute inflammatory QTL modulating neutrophil migration, interleukin 1 beta production, and protein concentration in the exudate using the F2 intercross of AIRmax and AIRmin mice [29], [30]. Additionally, our group detected the first QTL modulating PIA in mice, the *Prtia1* locus, located on chromosome 3, using mice selected for high and low antibody production [31]. The goal of the present work was therefore to discover new PIA QTL using the AIRmax and AIRmin mouse models, and to study the molecular basis underlying *Slc11a1 R and S* alleles effect in arthritis development through gene expression profiling within these QTL.

## Materials and Methods

### Mice

AIRmax and AIRmin lines (Ibut:AIRH and Ibut:AIRL formal stock designations at ILAR, Institute for Laboratory Animal Research, National Research Council), AIRmax*^RR^*, AIRmax*^SS^*, AIRmin*^RR^*, and AIRmin*^SS^* sublines, and F2 intercrosses were developed and maintained at the animal facilities of the Laboratory of Immunogenetics of the Butantan Institute. Male and female 8 to 12- week-old mice were used in the experiments. The F2 population was obtained by intercrossing AIRmax with AIRmin mice as described [29]. All procedures were approved by the Institutional Animal Care and Use Committee of the Butantan Institute.

### Pristane-induced Arthritis (PIA)

Mice received two intraperitoneal injections with 0.5 ml of the non-immunogenic mineral oil pristine (2,6,10,14-tetramethylpentadecane, Sigma Chemical Co., San Diego, USA) at 60-day intervals. Arthritis development was examined twice weekly for 160 days, by recording arthritis incidence and maximum severity scores for each paw. The severity scores, evaluated for each paw, were: 0–no signs of arthritis; 1–mild swelling of the toes or ankle joint; 2–moderate swelling; 3–severe swelling and/or ankylosis. The maximum score possible for any animal was 12. Phenotypes were assessed twice weekly by two independent observers and the animals were considered arthritic when the mean score assigned by the two observers was ≥2 [31].

### Genome-wide SNP Genotyping

Genomic DNA was extracted from tail tips using the E.Z.N.A.® Tissue DNA Kit (Omega Bio-Tek) and quantified using the Quant-iT™ PicoGreen®dsDNA Assay Kit (Invitrogen, Carlsbad, CA). SNPs were genotyped in F2 intercross mice utilizing the Bead-Array Platform (Illumina Inc. San Diego, CA), using the 1449-SNP loci mouse linkage panel as described [29]. Searches for QTL affecting the arthritis severity phenotype under study were carried out through genome-wide linkage analyses between genotypes and phenotypes by interval mapping using GridQTL version 3.1.0 [32] that uses a linear model to fit phenotype data according to genotypes. Additive and dominant effects at the QTL were included along with other explanatory variables of sex and family. The significance thresholds of phenotype-genotype associations were estimated by genome-wide permutation analysis.

### Affymetrix Microarray Analysis

Total RNA from the footpads of mice on days 0 and 160 after the second pristane injections were isolated using the RNAspin mini kit (GE Healthcare, Buckinghamshire, UK). The concentrations of the extracted RNA were checked using Nano-Drop (Thermo scientific), and their integrity was tested using Agilent 2100 Bioanalyzer (Agilent Technologies, Santa Clara, CA). The Affymetrix (Santa Clara, CA) Mouse Gene 1.0 ST Array was used, which is a whole transcript-based array that interrogates 28,853 well-annotated genes. Staining, hybridization, washing, and scanning of the array were performed following the manufacturers’ protocols at the AFIP-UNIFESP Molecular Facility, Federal University of Sao Paulo. Four experimental samples were run independently, providing replicates for each experimental group. Gene expression data was stored in a.cel format and subsequently exported to.txt files to be analyzed by Multi-viewer array software [33]. The data discussed in this publication have been deposited in NCBI’s Gene Expression Omnibus [34] and are accessible through GEO Series accession number GSE51516 (http://www.ncbi.nlm.nih.gov/geo/query/acc.cgi?acc=GSE51516).

### Real-time Quantitative PCR

Microarray results were validated by quantitative real-time PCR using gene-specific primers. Real-time PCR amplification mixtures contained 0.5 µl template cDNA, 12.5 µl SYBRGreen PCR Master Mix (Invitrogen, Carlsbad, CA, USA), and 0.3 µM specific PCR primers. Reactions were carried out in a Chromo 4 thermocycler (Bio-Rad, Hercules, CA). Targets and housekeeping genes were amplified with the primers described in Table 1. Relative differences were calculated according to the delta-delta Ct method [35]. Microarray data was correlated with qPCR results using Pearson’s analyses.

**Table 1:** qPCR versus Microarray results correlations and primers used.

Genes	Pearson (r)	Statistics	Foward	Reverse
***Il6***	0,91	p = 0,001	5′-GTTCTCTGGGAAATCGTGGA-3′	5′-TGTACTCCAGGTAGCTATGG-3′
***Il12a***	0,89	p = 0,001	5′-GATCATGAAGACATCACACGG-3′	5′-AGAATGATCTGCTGATGGTGG-3′
***Il10***	0,84	p = 0,01	5′-TCAAACAAAGGACCAGCTGGACAACATACTG-3′	5′-CTGTCTAGGTCCTGGAGTCCAGCAGACTCAA-3′
***Il1b***	0,81	p = 0,01	5′-CAGTTCTGCCATTGACCATC-3′	5′-TCTCACTGAAACTCAGCCGT-3′
***Tnfa***	0,82	p = 0,01	5′-TCTCATCAGTTCTATGGCCC-3′	5′-GGGAGTAGACAAGGTACAAC-3′
***Csf2***	0,80	p = 0,01	5′-TGAACCTCCTGGATGACATG-3′	5′-GTGTTTCACAGTCCGTTTCC-3′
***Cxcl1***	0.89	p = 0,001	5′- TGAAGCTCCCTTGGTTCAGA-3′	5′- AGGTGCCATCAGAGCAGTCT-3′
***Msr1***	0.91	p = 0,001	5′- GGGGAGTGTAGGCGGATCAACCCC-3′	5′- CGGCCCTCATGGGCTCCACTA-3′
***Hmox1***	0.87	p = 0,001	5′- ACCAGAGTCCCTCACAGATGGCG-3′	5′- GCAGGGGCAGTATCTTGCACCA-3′
***β2microglubulin***	0.97	p = 0,001	5′TGACCGGCTTGTATGCTATC-3′	5′-CAGTGTGAGCCAGGATATAG-3′

Relative differences were calculated according to the delta-delta Ct method. Microarray data was correlated with qPCR results using Pearson’s analyses and the same four samples on each line.

### Statistical Analyses

The gene expression observed in each array was log-transformed to approximate a Gaussian distribution and then standardized over the array to adjust for systematic differences in their expressions. Differentially expressed genes were detected using Significance of Analysis of Microarray (SAM) software (Two class unpaired, FDR ≤5%) that examined the data from four biological replicates on each line [36]. We analyzed all of the significant differentially-expressed genes for the over-represented biological themes using Expression Analysis Systematic Explorer (EASE) software [37]. This program automates the process of biological theme determination using Gene Ontology (GO) classification. EASE calculates over-representation with respect to the total number of genes assayed and annotated within each system, allowing for side-by-side comparisons of categories from categorization systems with varying levels of annotation. EASE will thus rapidly convert lists of genes into an ordered table of robust biological themes. Calculating statistics on thousands of gene categories can, however, lead to a few seemingly significant probabilities simply due to chance. To address this multiple comparison issue, we used a Bonferroni-type probability correction.

## Results

### QTL Detection

**Figure 1:**
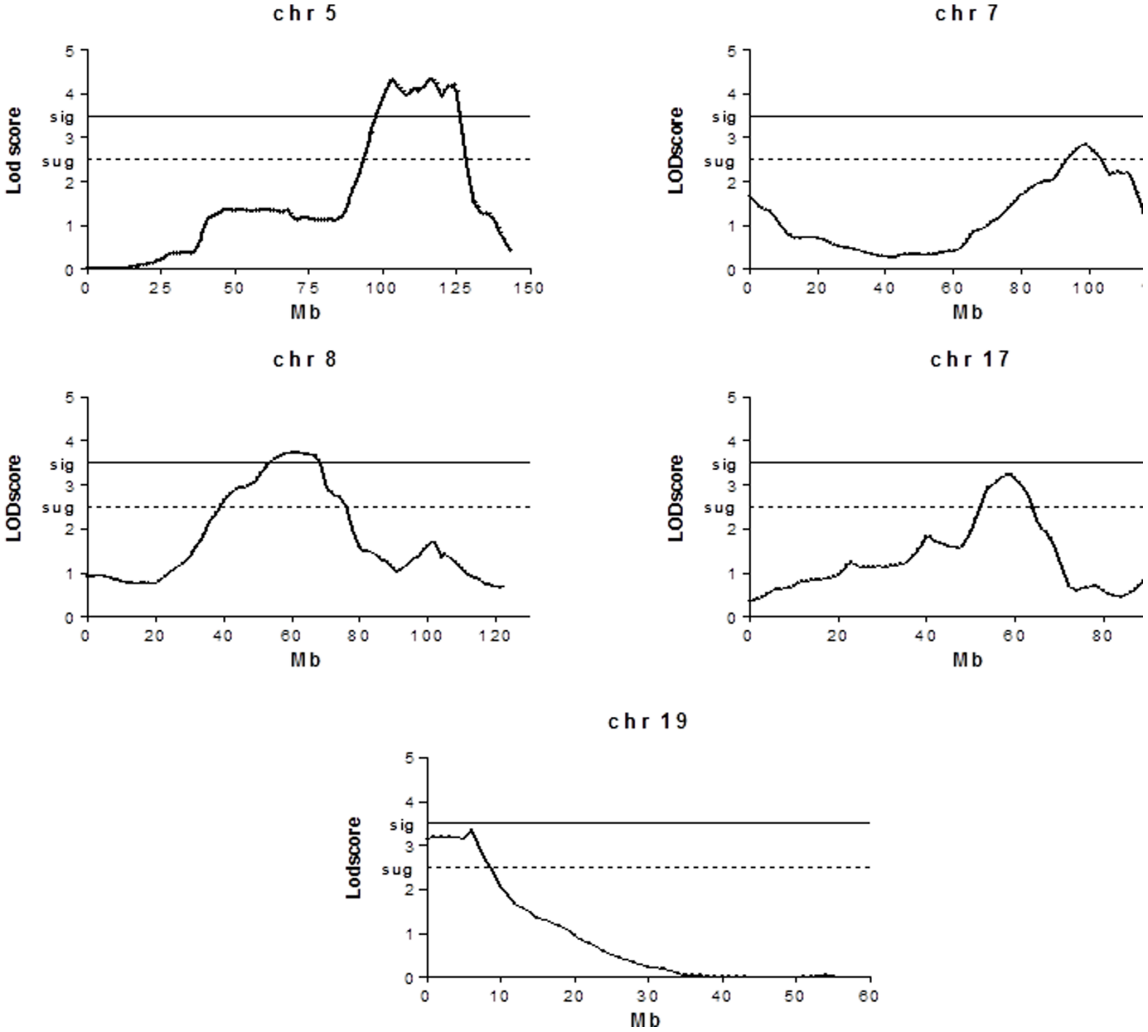
Mapping of the QTL regulating PIA severity phenotype in mice selected for acute inflammation. SNPs were genotyped in F2 intercrosses employing the Bead-Array Platform (Illumina Inc. San Diego, CA) and using the 1449-SNP loci mouse linkage panel as described. Searches for QTL affecting the arthritis severity phenotype under study were carried out using genome-wide linkage analysis between genotypes and phenotypes by interval mapping, using GridQTL version 3.1.0.

Genome-wide scanning was performed in 290 F2 intercross mice with the PIA severity scores phenotype. Figure 1 shows PIA severity quantitative trait loci (arthritis QTL) presenting high linkage significance (LODscore>4) on chromosomes 5 and 8, and several suggestive QTL on chromosomes 7, 17 and 19. The 1 LOD score confidence interval (CI) on Chromosome 5 maps from 95 to 125 Mb while on chromosome 8 the CI ranges from 40 to 85 Mb, considering LOD scores of 4.177 and 3.865 respectively. The three other suggestive QTL showed wide CI, with LOD scores around 3.

### Global Gene Expression and qPCR Analysis

**Figure 2:**
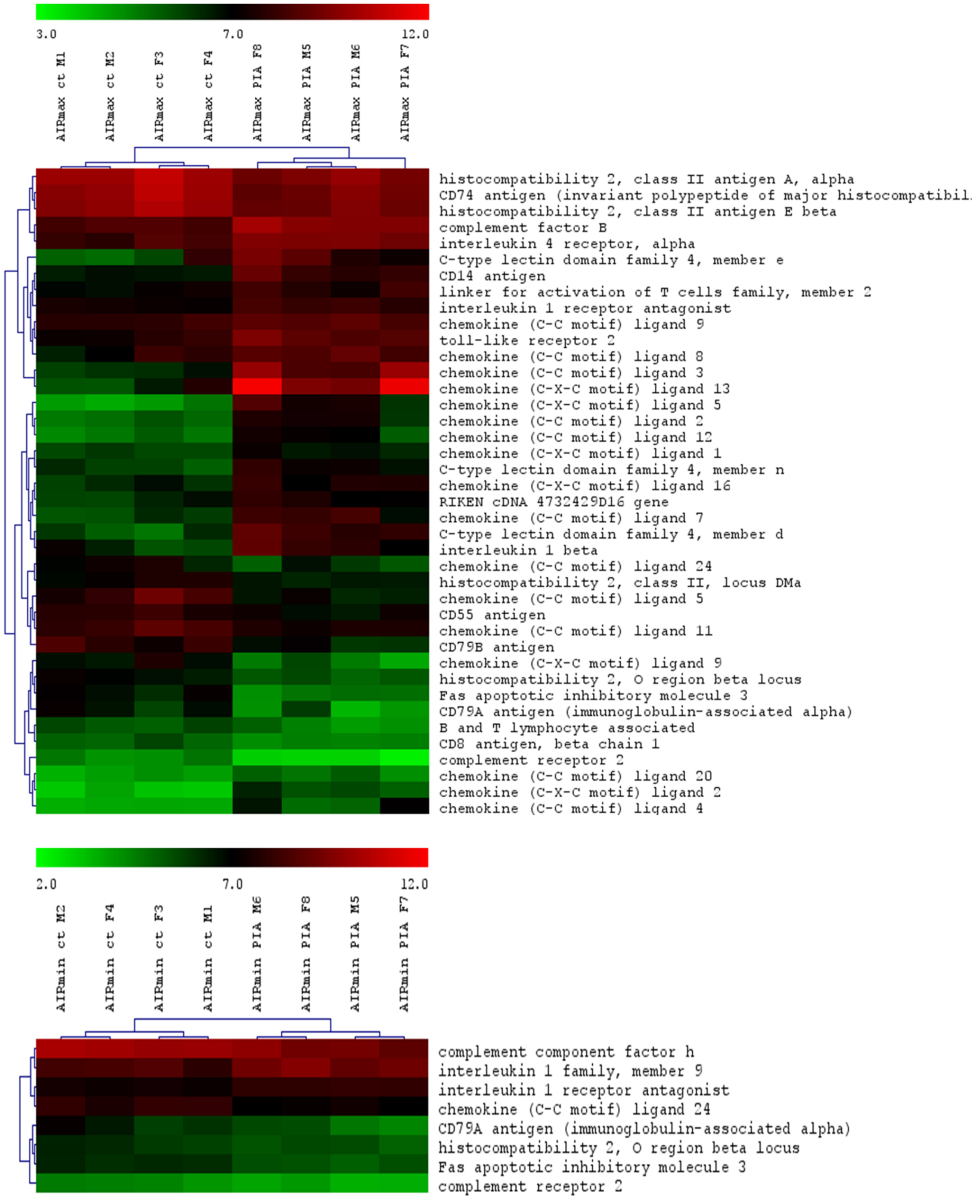
Up- and down-modulated inflammatory and chemokine genes in AIRmax and AIRmin mice. Mouse Gene 1.0(SAM) software (Two class unpaired, FDR ≤5%) that examined the data from four biological replicates on each line.

**Figure 3:**
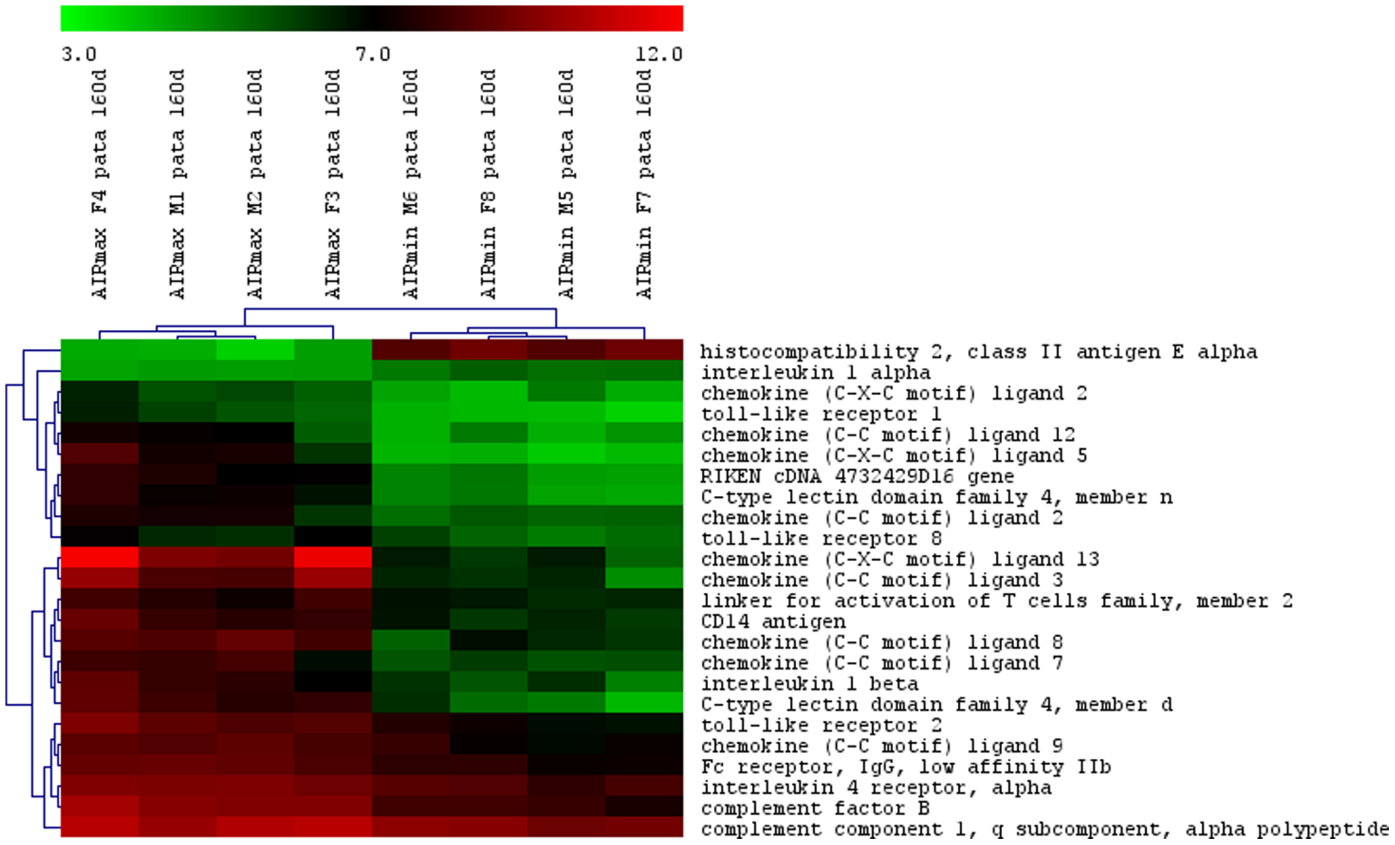
Differentially expressed inflammatory and chemokine genes in AIRmax and AIRmin mice. Mouse Gene 1.0(SAM) software (Two class unpaired, FDR ≤5%) that examined the data from four biological replicates on each line.

**Figure 4:**
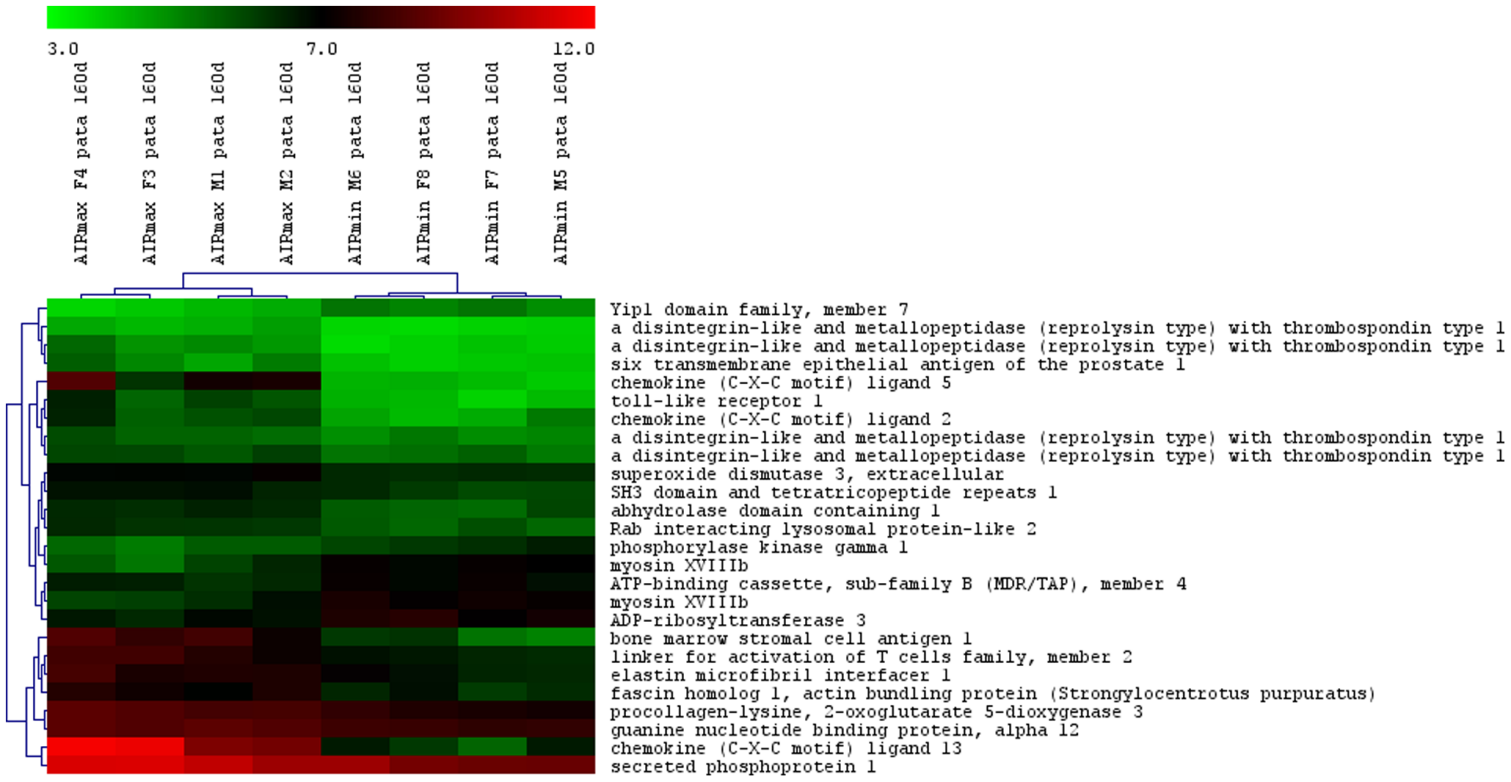
Differentially expressed genes on chromosome 5 between AIRmax and AIRmin mice. Mouse Gene 1.0(SAM) software (Two class unpaired, FDR ≤5%) that examined the data from four biological replicates on each line.

**Figure 5:**
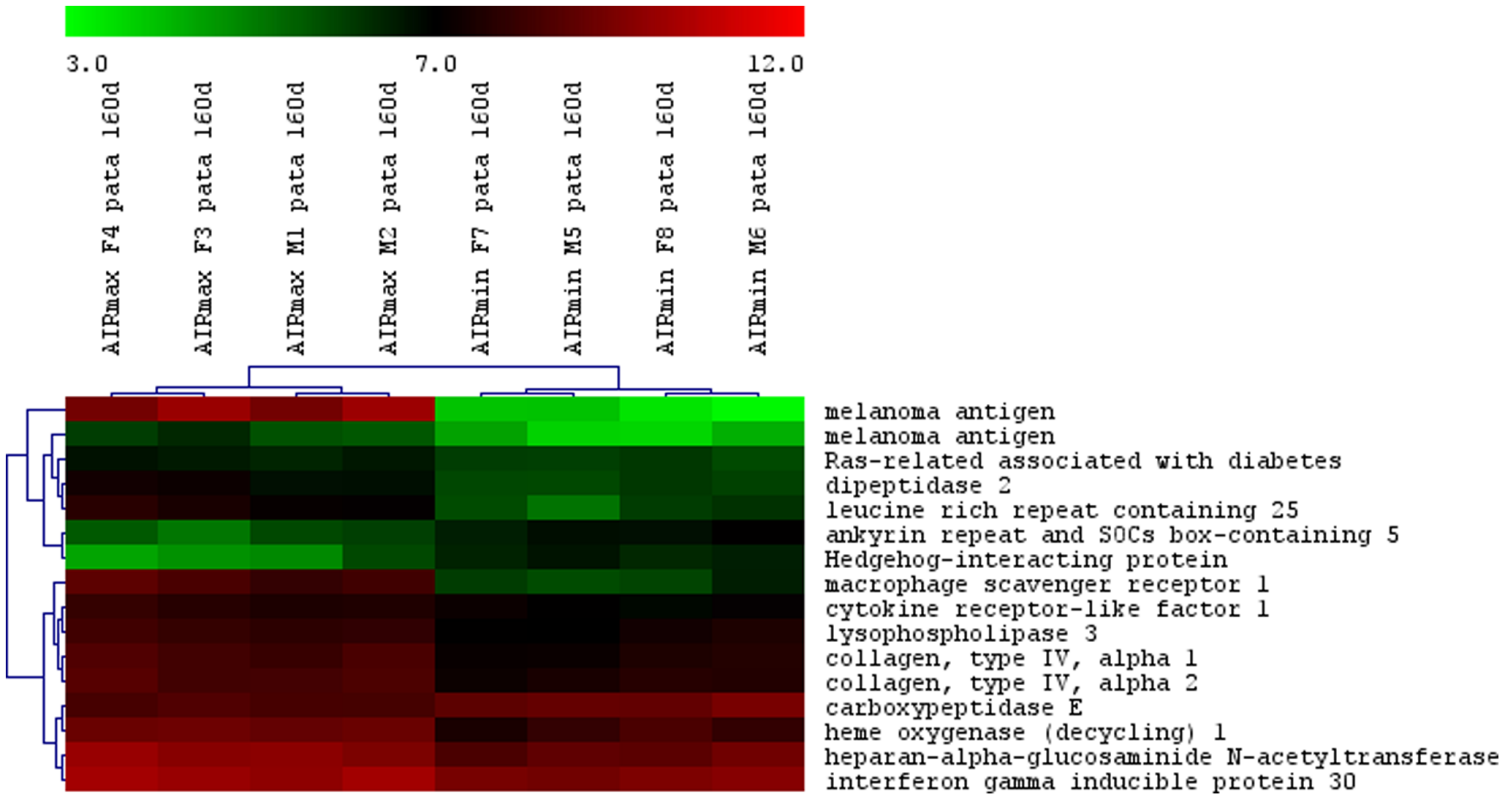
Differentially expressed genes on chromosome 8 in AIRmax and AIRmin mice. Mouse Gene 1.0(SAM) software (Two class unpaired, FDR ≤5%) that examined the data from four biological replicates on each line.

**Figure 6:**
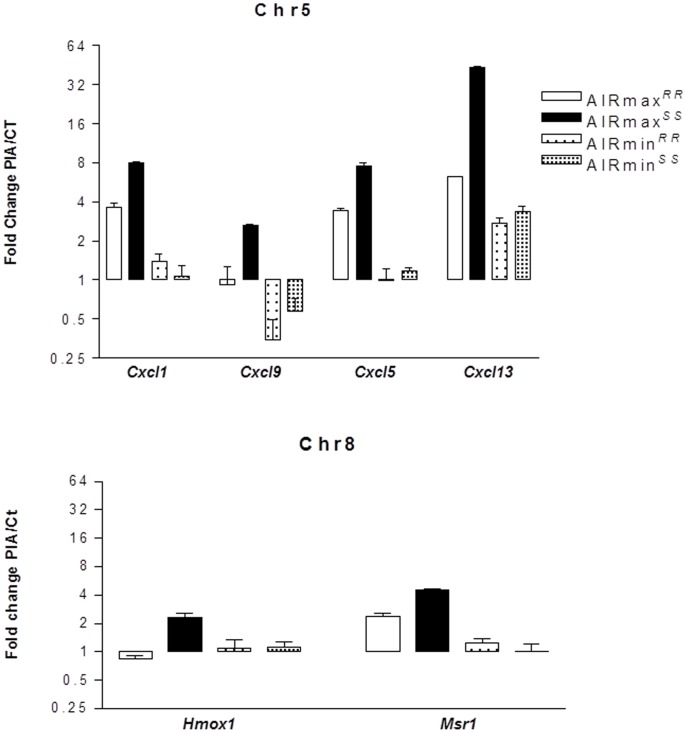
Signal intensities of differentially-expressed genes from AIRmax and AIRmin mouse sublines. Mouse Gene 1.0(SAM) software (Two class unpaired, FDR ≤5%) that examined the data from four biological replicates on each line.

The total numbers of the up- and down-modulated inflammatory response genes are distinct in each line, being higher in AIRmax than in AIRmin mice (Figure 2). These genes are expressed in the swelled footpad of the mice, where there is a large infiltrate of inflammatory neutrophils and macrophages. Global gene expression analysis indicated 419 differentially expressed genes between AIRmax and AIRmin mice. EASE analysis demonstrated significantly over-represented genes (P<0.001) related to inflammatory responses and chemotaxis (Figure 3). Figures 4 and 5 show genes differentially expressed on the confidence intervals of the PIA QTLs at chromosomes 5 and 8 respectively. Several genes related to inflammation, cell adhesion, and chemotaxis could be observed on chromosome 5, while tissue antigens, cell differentiation, hemeoxigenase and scavenger receptor genes were observed on chromosome 8. AIRmax and AIRmin *Slc11a1* sublines showed distinct gene expression profiles, reinforcing these results. Higher up-regulation of the chemokine genes *Cxcl1, Cxcl9, Cxcl5, Cxcl13* on chromosome 5 were observed in AIRmax*^SS^* than in the other lines. The *macrophage scavenger receptor 1*(*Msr1*) and *hemeoxigenase (decycling) 1*(*Hmox1*) genes on chromosome 8, were also more actively expressed in AIRmax*^SS^*mice (Figure 6). High correlations among qPCR and Microarray results can be seen in Table 1, indicating the validity of these experiments. These results revealed two significant arthritis QTL (on chromosomes 5 and 8) interacting with the *Slc11a1* gene to create a gene expression profile that contributes to the enhanced susceptibility of AIRmax*^SS^* mice to PIA.

## Discussion

The present work mapped two new PIA QTL (*Prtia* 2 and *Prtia*3), on chromosomes 5 and 8, respectively. Three suggestive QTL were detected on chromosomes 7, 17 and 19. The first PIA loci detected in mice was *Prtia* 1 (by our group) on chromosome 3 using animals selected for high and low antibody production [31]. We also described the involvement of *Slc11a1* alleles in PIA using the present AIRmax and AIRmin mouse model [5].

We have recently mapped loci that regulate the intensity of the acute inflammatory response on chromosomes 5, 7, 8 and 17, which overlap the newly detected PIA QTL, suggesting common regulations [29], [30]. Co-located chromosome 5 QTL controlling arthritis severity and humoral responses during *B. burgdorferi* infection were identified in the F2 intercross of C3H/HeNCr and C57BL/6NCr mice [38], confirming the involvement of the chemokine *Cxcl9* gene in this model [39]. QTL on chromosomes 5 and 8 were also detected in two other arthritis models (collagen and proteoglycan induced arthritis) [40], suggesting the importance of these related gene clusters to arthritis susceptibility.

Regarding the suggestive QTL detected in this work, the loci on chromosomes 7, 17 and 19 overlap regions detected for acute inflammation where map inflammasome/integrins, MHC and ion transport related genes, respectively (Table 2) [30]. AIRmax and AIRmin mice present distinct MHC haplotypes: AIRmax H2b and AIRmin H2kd [1]. *H2Ea* gene expression was low in AIRmax mice (Figure 3), in agreement with a report describing a correlation of the H2^b^ haplotype with low *H2Ea* gene expression [41].

**Table 2:** Differentially-expressed major candidate genes detected in microarray related to PIA QTL.

*Genes*	*Chromosomes*	*AIRmax/* *AIRmin ratio*	*GO Biological process*
*Slc11a1*	1	2.98	Transport
*Cxcl1*	5	3.78	Chemokine
*Cxcl9*	5	2.67	Chemokine
*Cxcl5*	5	12.11	Chemokine
*Cxcl13*	5	27.50	Chemokine
*Saa3*	7	21.39	Inflammation
*Il21r*	7	2.82	Inflammation
*Bcl3*	7	2.03	Apoptosis
*Msr1*	8	5.02	Scavenger
*Hmox1*	8	2.05	Angiogenesis/apoptosis
*H2ea*	17	0.03	Antigen Presentation
*Slc15a3*	19	2.55	Transport

Differentially expressed genes in AIRmax and AIRmin mice. Mouse Gene 1.0 ST Array was used. Differentially expressed genes were detected using Significance of Analysis of Microarray (SAM) software (Two class unpaired, FDR ≤5%) that examined the data from four biological replicates on each line.

The total number of up- and down-regulated genes in each line was distinct, as can be seen in Figure 2. More genes were modulated in AIRmax than in AIRmin mice, although we observed an over-representation of genes related to inflammatory reactions and chemotaxis (gene ontology) biological themes in both lines. This same gene expression profile was observed in bone marrow cells from these lines after Biogel stimulus [42], indicating the generalized action of selective pressure during the phenotype selection process.

Ibrahim and collaborators investigated the gene expression profiles of inflamed paws in DBA1 inbred mice using a similar approach for collagen-induced arthritis [43]. In that work, inflammation resulted in increased gene expression of matrix metalloproteinases, and immune-related extra-cellular matrix and cell-adhesion molecules, as well as molecules involved in cell division and transcription, in a manner very similar to our model. However, the total number of differentially-expressed genes involved in the inbred mouse model (223) was lower than in our model (419), suggesting that the heterogeneous background of AIRmax and AIRmin mice permitted a larger genome involvement in this phenotype.

The high acute inflammation observed in AIRmax mice appears to result from the accumulation of three convergent elements during their selection: 1) higher numbers of neutrophils in their bone marrow, as a consequence of an elevated response to granulopoietic cytokines; 2) high concentrations of chemotatic factors in their exudates; and, 3) strong resistance of their neutrophils to spontaneous apoptosis [7]. Consistent with these alterations, AIRmax mice are more susceptible to arthritis [1], LPS shock [6], and colon carcinogenesis [44]. However, this high-responder line demonstrated extreme resistance to bacterial infections [3] as well as to lung [45] and skin carcinogenesis [2]. Neutrophils are the first cells to be recruited to a damaged site [46], and CXC chemokines (including CXCL2 and CXCL1) are the most critical inflammatory mediators for this recruitment [47]. Among the differentially-expressed genes observed in the present work, inflammatory and chemokine genes on chromosome 5 and *macrophage scavenger receptor 1* (*Msr1*) and *hemeoxigenase 1* (*Hmox1*) genes on chromosome 8 appear to be the major candidates.

Chemokines are involved in leukocyte recruitment to inflammatory sites, such as to synovial tissue in rheumatoid arthritis (RA). However, they may also be homeostatic as these functions often overlap [48]. Chemokines have essential roles in the recruitment and activation of leucocyte subsets within tissue microenvironments, and stromal cells actively contribute to these networks. It has been demonstrated that inappropriate constitutive chemokine expression contributes to the persistence of inflammation by actively blocking its resolution [49]. This was likewise observed in lung carcinogenesis in our model, as transcriptome analysis revealed that the genes involved in transendotelial migration and chemokine-cell adhesion were differently-expressed in normal lungs of susceptible AIRmin and resistant AIRmax mice [50], suggesting important roles for these phenotypes in chronic diseases.

Macrophages play a central role in the pathogenesis of rheumatoid arthritis (RA), which is marked by an imbalance of inflammatory and anti-inflammatory macrophages in RA synovium. Although the polarization and heterogeneity of macrophages in RA have not been fully elucidated, the identities of macrophages in RA can potentially be defined by their products, including co-stimulatory molecules, scavenger receptors, different cytokines/chemokines and receptors, and transcription factors. Efforts have been made to understand polarization, apoptosis regulation, and the novel signaling pathways in macrophages [51]. Serum from rheumatoid arthritis patients (but not from healthy subjects) increased mRNA expression of the *Msr1* gene in macrophages. Human arterial endothelial cells are inhibited by both anti-IL-6 receptor antibodies (α-IL-6R Ab) and TNF-α receptors (p75)-Fc (TNFR-Fc), suggesting an important role for this receptor in chronic inflammatory disease [52].

Heme oxygenase-1 has potent antioxidant and anti-inflammatory functions, although the underlying mechanisms are not yet well understood [53]. The disruption of *Hmox1* in macrophages *in vivo* and the release of nonmetabolized heme cause tissue inflammation [54]. Additionally, expression of the *Slc11a1* gene has been associated with a twofold increase in *Hmox1* transcriptional levels in macrophages undergoing erythrophagocytosis [55]. Although *Hmox1* seems to alter macrophage activity, no involvement with arthritis is described in the literature. In the present work, AIRmax*^SS^* mice homozygous for the *Slc11a1 S* allele showed higher *Hmox1* and *Msr1* gene expression than AIRmax*^RR^* mice, suggesting that *Slc11a1* interacts with these two genes (figure 4). Similarly, chemokine genes are also up-regulated in AIRmax*^SS^* mice, evidencing a specific gene expression profile of this line that favors chronic inflammation and consequent susceptibility to arthritis. In this way, *Slc11a1 S* allele should alter the macrophage activity, promoting modulation of chemokines, heme oxygenase, apoptosis, transport, scavenger receptor and inflammatory genes expressed by these cells (Table 2).

The interaction of the *Slc11a1 S* allele with high inflammatory background QTL in AIRmax mice modulated the Biogel-induced early acute inflammation, infection resistance [6], and pristane induced-arthritis [5]. AIRmax*^SS^* also showed faster ear-wound closure than AIRmax*^RR^* mice, suggesting that the *Slc11a1S* allele favors ear tissue regeneration [8].

Taken together, the results of the present work provide interesting insights into the molecular inflammatory mechanisms involved in arthritis onset, and may also exert influences on other experimentally induced diseases. Further studies will be necessary to gain a thorough understanding of the genetic and cellular mechanisms involved in arthritis progression and to provide models for investigating therapeutic treatments that could modulate inflammatory events.
