# Correction: Pristane-Induced Arthritis Loci Interact with the *Slc11a1* Gene to Determine Susceptibility in Mice Selected for High Inflammation

**DOI:** 10.1371/journal.pone.0092525

**Published:** 2014-03-10

**Authors:** The PLOS ONE Staff

The name of the 12^th^ author is incorrectly given in the author byline. The correct name is: Tommaso A. Dragani.
